# Supplementary material for: Multilocus Phylogeography and Population Genetic Analyses of Opsariichthys hainanensis Reveal Pleistocene Isolation Followed by High Gene Flow around the Gulf of Tonkin
Source: Genes (Basel). 2022 Oct 20;13(10):1908. doi: 10.3390/genes13101908 (PMC9602001; doi:10.3390/genes13101908)
Supplement: Supplementary file 1 [file genes-13-01908-s001.zip › genes-1919844-supplementary.pdf]

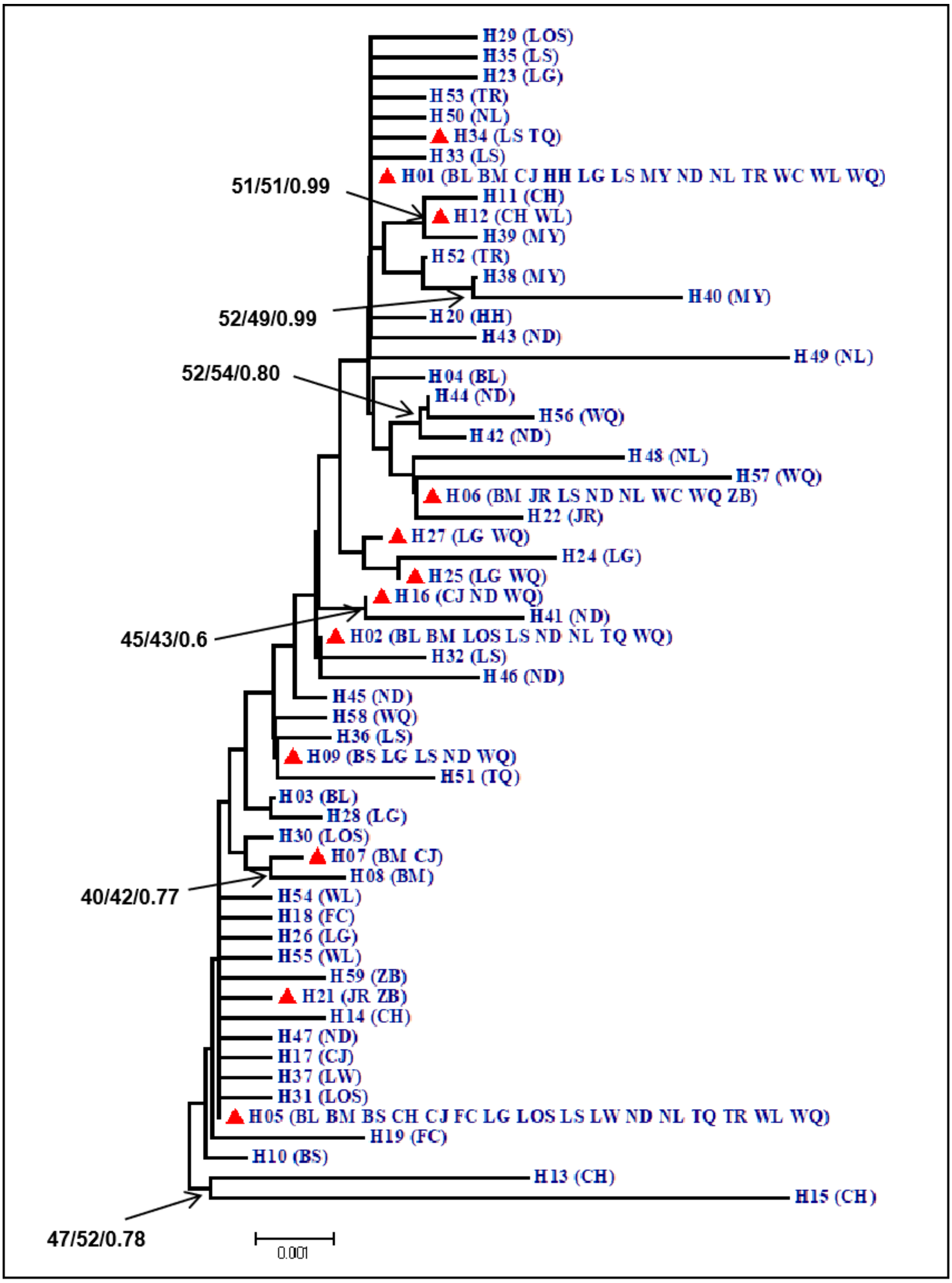

Figure S1. NJ tree of genetic relationships based on nuclear RAG1 gene among 21 populations in *Opsariichthys hainanensis* using 59 haplotypes. Red triangles mean shared haplotype.

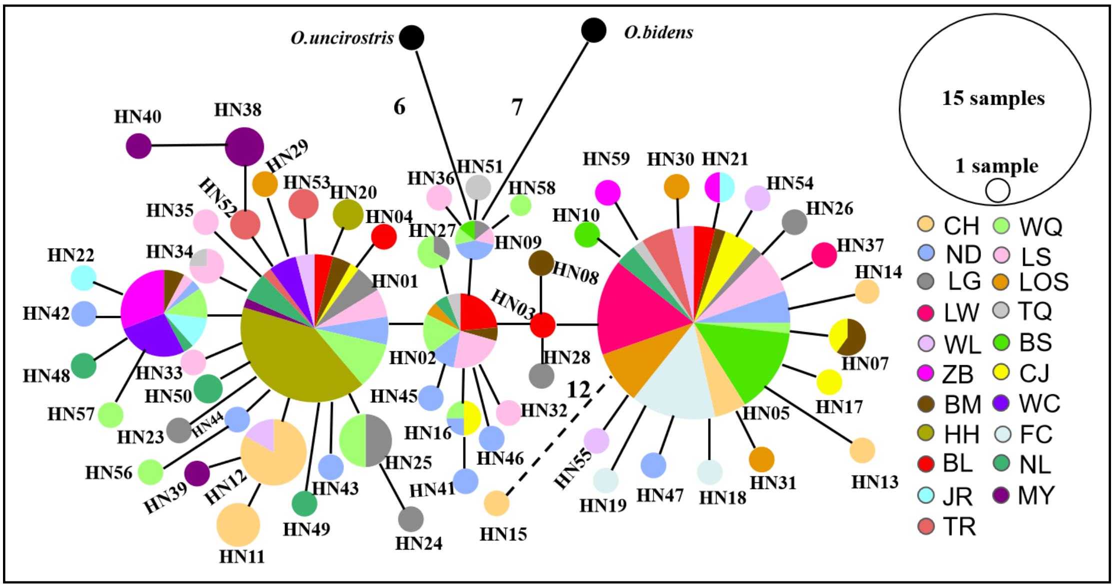

Figure S2. Minimum spanning network (MSN) based on mutations between haplotypes observed in populations of *Opsariichthys hainanensis* based on nuclear RAG1 gene. Haplotype designations (Table 1) are indicated next to each circle. Locality designations (see Figure 1) for specimens possessing each haplotype are indicated inside the circles. The sizes of the circles are proportional to the number of individuals represented. The length of the lines between circles is roughly proportional to the estimated number of mutational steps between the haplotypes. *Opsariichthys bidens* and *O. uncirostris* are two species in the genus Opsariichthys used as an out-group.

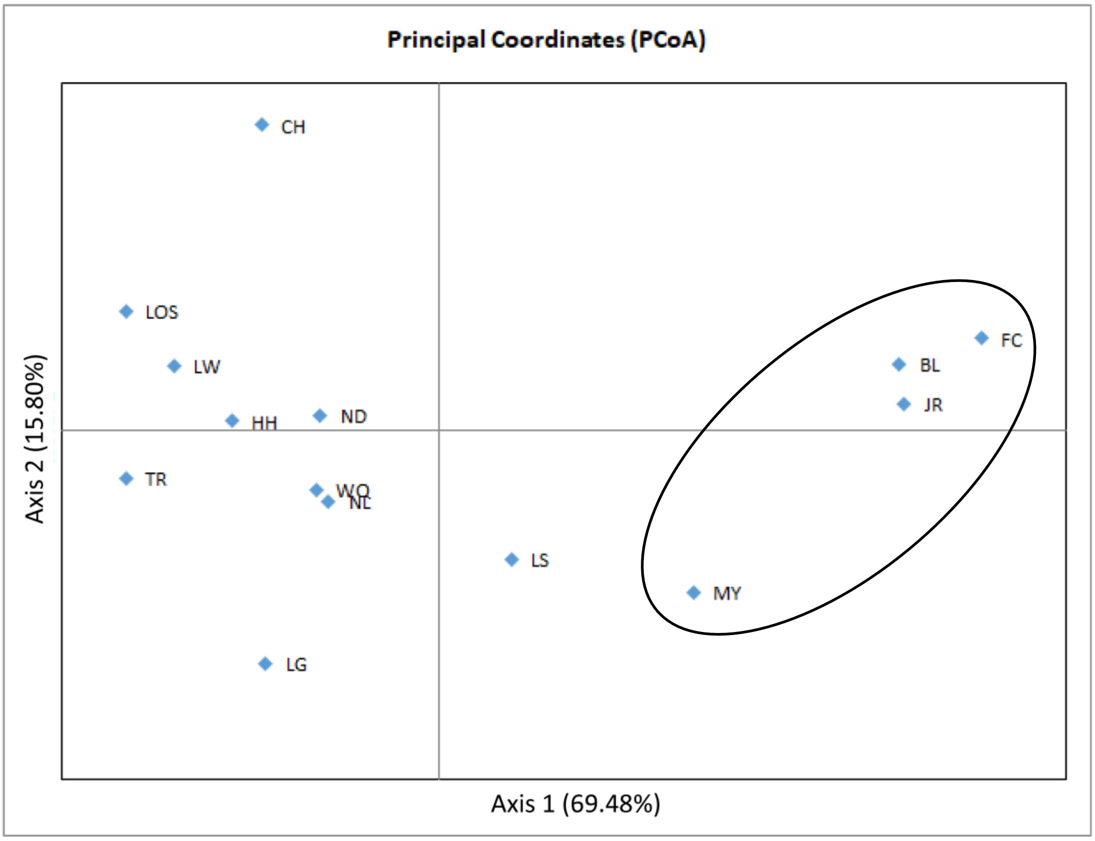

Figure S3. Plot of principal coordinate (PCA) axes for fourteen populations of *Opsariichthys hainanensis* based on population genetic distance. Percentage values represent variation justified by each axis. See Table 1 for the sampling sites/population codes. Circle mark the same group.

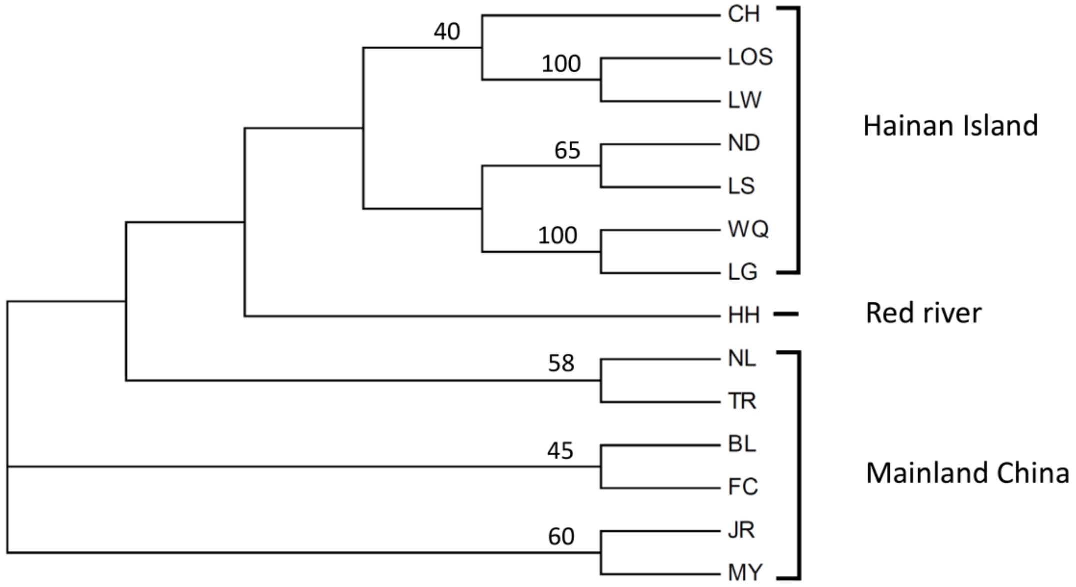

Figure S4. A neighbour-joining tree reconstructed based on the Cavalli-Sforza and Edwards genetic distance (Dc) among fourteen populations in *Opsariichthys hainanensis* based on microsatellite DNA. See Table 1 for the sampling sites/population codes.

Table S1 Matrix of pairwise F_{ST} among twenty-one populations based on mtDNA (below diagonal) and nuclear RAG-1 gene (above diagonal) in *Opsariichthys hainanensis*.

	CH	ND	WQ	LS	LG	LOS	LW	TQ	WL	BS	ZB	CJ	BM	WC	HH	BL	FC	NL	JR	MY	TR
CH		0.196	0.213	0.186	0.207	0.310	0.445	0.162	0.085	0.423	0.261	0.282	0.212	0.295	0.282	0.185	0.432	0.134	0.223	0.391	0.147
ND	0.722		0.072	-0.007	0.119	0.146	0.359	-0.029	0.020	0.317	0.155	0.086	0.033	0.265	0.303	-0.022	0.340	0.023	0.125	0.455	0.025
WQ	0.464	0.511		0.068	0.011	0.356	0.549	0.131	0.150	0.518	0.153	0.292	0.159	0.179	0.172	0.090	0.528	0.031	0.131	0.439	0.102
LS	0.686	0.173	0.487		0.103	0.212	0.440	-0.030	0.024	0.400	0.215	0.155	0.072	0.312	0.266	-0.030	0.416	0.016	0.191	0.472	0.012
LG	0.478	0.519	-0.023	0.481		0.333	0.545	0.129	0.127	0.512	0.259	0.285	0.178	0.333	0.289	0.121	0.512	0.066	0.226	0.462	0.095
LOS	0.730	0.923	0.511	0.888	0.585		0.062	0.094	0.076	0.018	0.430	-0.047	0.115	0.666	0.767	0.223	0.045	0.219	0.395	0.663	0.191
LW	0.731	0.923	0.511	0.888	0.585	-0.042		0.471	0.360	0.000	0.691	0.169	0.386	0.912	0.959	0.578	0.000	0.444	0.718	0.834	0.496
TQ	0.638	0.666	0.422	0.399	0.393	0.970	0.973		-0.029	0.391	0.268	0.040	0.030	0.524	0.650	0.000	0.372	0.003	0.179	0.520	0.006
WL	0.693	0.668	0.481	0.553	0.486	0.987	0.990	0.887		0.306	0.253	0.050	0.025	0.453	0.508	0.242	0.304	0.022	0.202	0.504	-0.021
BS	0.685	0.714	0.505	0.602	0.513	0.970	0.972	0.815	0.868		0.658	0.117	0.343	0.882	0.937	0.518	0.000	0.407	0.675	0.812	0.445
ZB	0.677	0.581	0.455	0.490	0.456	0.965	0.967	0.756	0.852	0.827		0.345	0.177	0.028	0.517	0.303	0.638	0.067	-0.113	0.541	0.223
CJ	0.628	0.348	0.408	0.284	0.386	0.917	0.919	0.481	0.633	0.648	0.287		-0.010	0.586	0.712	0.147	0.136	0.164	0.303	0.623	0.145
BM	0.644	0.036	0.408	0.138	0.386	0.928	0.930	0.592	0.671	0.696	0.514	0.187		0.377	0.521	0.066	0.342	0.051	0.135	0.525	0.057
WC	0.688	0.350	0.472	0.344	0.477	0.986	0.988	0.895	0.945	0.894	0.861	0.611	0.376		0.694	0.511	0.846	0.122	0.058	0.651	0.398
HH	0.666	0.837	0.576	0.799	0.626	0.918	0.919	0.862	0.887	0.878	0.874	0.820	0.827	0.883		0.529	0.906	0.153	0.599	0.727	0.407
BL	0.690	0.691	0.516	0.586	0.524	0.973	0.976	0.838	0.868	0.837	0.822	0.646	0.676	0.897	0.880		0.502	0.021	0.283	0.565	0.031
FC	0.665	0.512	0.445	0.380	0.438	0.954	0.956	0.647	0.767	0.764	0.477	0.211	0.413	0.785	0.856	0.751		0.413	0.639	0.789	0.433
NL	0.688	0.671	0.491	0.569	0.496	0.964	0.966	0.799	0.828	0.713	0.791	0.613	0.642	0.864	0.834	0.831	0.716		0.016	0.354	-0.006
JR	0.668	0.514	0.448	0.411	0.446	0.974	0.977	0.818	0.875	0.834	0.782	0.532	0.510	0.848	0.875	0.829	0.678	0.801		0.519	0.177
MY	0.684	0.568	0.469	0.460	0.473	0.978	0.980	0.844	0.899	0.859	0.813	0.584	0.566	0.878	0.881	0.857	0.723	0.825	0.711		0.422
TR	0.669	0.458	0.434	0.349	0.433	0.993	0.995	0.904	0.965	0.889	0.838	0.523	0.490	0.943	0.883	0.891	0.713	0.850	0.568	0.792	

Table S2. Characteristics and genetic diversity indices for fourteen populations in *Opsariichthys hainanensis*.

locations (Abbreviation)	Sample size (N)	Number of allele (N_A)	Observed heterozygosity (H_O)	Expected heterozygosity (H_E)	Allele richness (A_R)	Inbreeding coefficient (F_{IS})
**Hainan Island**	**150**	**7.441**	**0.527**	**0.716**	**5.489**	**0.264**
Changhua River (CH)	27	9.917	0.600	0.815	6.812	0.267
Nandu River (ND)	30	9.333	0.550	0.762	6.091	0.281
Wanqan River (WQ)	30	10.008	0.576	0.787	6.332	0.271
Lingshui River (LS)	30	7.917	0.478	0.735	5.536	0.354
Longgun River (LG)	15	6.167	0.533	0.705	5.082	0.250
Longshou River (LOS)	8	5.167	0.479	0.663	5.167	0.291
Longwei River (LW)	10	3.583	0.475	0.546	3.400	0.136
**mainland China**	**114**	**6.179**	**0.524**	**0.656**	**4.892**	**0.209**
Yuanjiang River (HH)	30	10.667	0.559	0.774	6.681	0.281
Beilun River (BL)	15	4.583	0.406	0.544	3.772	0.262
Fangheng River (FC)	15	3.333	0.402	0.518	3.023	0.229
Nanliu River (NL)	14	6.917	0.649	0.746	5.693	0.135
Jian River (JR)	15	4.750	0.461	0.552	3.833	0.170
Moyang River (MY)	15	6.833	0.525	0.676	5.554	0.230
Tan River (TR)	10	6.167	0.664	0.779	5.685	0.154
**Total**	**264**	**6.81**	**0.526**	**0.686**	**5.190**	**0.237**

Table S3 Characteristics and genetic diversity indices for twelve microsatellite loci in *Opsariichthys hainanensis*.

Locus		Primer sequences	Number of alleles (N_a)	Allelic richness (A_R)	Observed heterozygosity (H_O)	Expected heterozygosity (H_E)	Inbreeding coefficient (F_{IS})
Loci-1	12715_23_TC-F	5’-TGTAAAACGACGGCCAGTACACAGCAGCCATCTCAATCT-3’	16	7.039	0.309	0.499	0.390
	12715_23_TC-R	5’-CGGAACTCGCTCTTTCTCTCT-3’					
Loci-2	66209_12_CA-F	5’-TGTAAAACGACGGCCAGTTTTGGACCGTTTCCTCCTTAC-3’	21	8.697	0.319	0.677	0.537
	66209_12_CA-R	5’-AATGGTTACAGTGAGTGATGCT-3’					
Loci-3	19558_19_CA_F	5’-TGTAAAACGACGGCCAGTCCGACCAATCAGAGCACAGT-3’	23	9.256	0.612	0.674	0.097
	19558_19_CA_R	5’-CTTCGACTTACGACATGACCTT-3’					
Loci-4	46702_25_AC-F	5’-TGTAAAACGACGGCCAGTCTTGTCCTTCACTGTTGTCACT-3’	19	8.725	0.614	0.746	0.182
	46702_25_AC-R	5’-GGCTGCTACACTTGCTACTAAT-3’					
Loci-5	29014_21_GT_F	5’-TGTAAAACGACGGCCAGTCCAGATTGGACCAGTAGTTCAA-3’	25	9.743	0.600	0.756	0.213
	29014_21_GT_R	5’-TTCTACAAGCCAGCACAGAC-3’					
Loci-6	47708_24_CA-F	5’-TGTAAAACGACGGCCAGTAGGCATTACTGATTAGGCACAA-3’	28	9.116	0.611	0.732	0.169
	47708_24_CA-R	5’-ACATCGTTATGGCAGAACACAA-3’					
Loci-7	16991_11_CA-F	5’-TGTAAAACGACGGCCAGTGTAGAATCGTGTCGCTGTAATG-3’	12	5.381	0.407	0.534	0.246
	16991_11_CA-R	5’-CTGGGAGCAGGACAGTAAATAC-3’					
Loci-8	36185_19_GT_F	5’-TGTAAAACGACGGCCAGTATGCCTATGGAATGTCCTCACT-3’	21	8.816	0.455	0.732	0.386
	36185_19_GT_R	5’-GACTCATTACGCCAGCACAG-3’					
Loci-	51469_19_GT-F	5’-TGTAAAACGACGGCCAGTCACCAGGCAACAAGGAAGTC-3’	16	6.401	0.508	0.651	0.225

9	51469_19_GT-R	5'-ATGTGTTCACTCAACTCCAAGT-3'					
Loci-10	69621_12_TG-F	5'-TGTAAAACGACGGCCAGTGCTCCGAGACTCTGACACA-3'	13	6.602	0.520	0.668	0.228
	69621_12_TG-R	5'-CGCAAGACGAGAAGTAACAATG-3'					
Loci-11	57732_19_AG-F	5'-TGTAAAACGACGGCCAGTGTCTCCTTGGCAACAATAACTG-3'	23	9.503	0.687	0.781	0.124
	57732_19_AG-R	5'-CACATTCCTCCTCATCCTCAC-3'					
Loci-12	62825_28_AG-F	5'-TGTAAAACGACGGCCAGTGCTTCATGGTGGCTGTAGAG-3'	30	10.711	0.664	0.782	0.157
	62825_28_AG-R	5'-TGAATGCTGAGTCCAGTATTGA-3'					
Mean			20.583	8.332	0.526	0.686	0.241

Table S4 Matrix of pairwise F_{ST} (below diagonal) and R_{ST} (above diagonal) among fourteen populations based on microsatellite DNA in *Opsariichthys hainanensis*.

Population	CH	ND	WQ	LS	LG	LOS	LW	HH	BL	FC	NL	JR	MY	TR
CH		0.174	0.157	0.153	0.171	0.373	0.415	0.399	0.343	0.467	0.202	0.434	0.323	0.365
ND	0.086		0.144	0.088	0.142	0.478	0.557	0.379	0.393	0.484	0.159	0.450	0.436	0.354
WQ	0.103	0.085		0.116	0.137	0.385	0.485	0.325	0.351	0.465	0.018	0.478	0.414	0.322
LS	0.111	0.057	0.101		0.045	0.243	0.314	0.393	0.276	0.303	0.130	0.280	0.342	0.267
LG	0.123	0.090	0.038	0.120		0.269	0.377	0.367	0.390	0.348	0.160	0.367	0.369	0.352
LOS	0.146	0.182	0.194	0.188	0.244		0.048	0.481	0.640	0.527	0.459	0.639	0.506	0.621
LW	0.202	0.243	0.263	0.254	0.318	0.007		0.563	0.692	0.615	0.558	0.694	0.564	0.691
HH	0.131	0.138	0.124	0.150	0.133	0.223	0.283		0.429	0.460	0.277	0.498	0.343	0.281
BL	0.250	0.262	0.260	0.258	0.318	0.372	0.419	0.305		0.585	0.367	0.420	0.282	0.272
FC	0.258	0.255	0.265	0.258	0.305	0.387	0.435	0.255	0.412		0.531	0.620	0.569	0.594
NL	0.113	0.121	0.134	0.109	0.157	0.223	0.294	0.156	0.226	0.259		0.526	0.385	0.262
JR	0.229	0.207	0.195	0.201	0.219	0.348	0.406	0.266	0.346	0.378	0.201		0.240	0.417
MY	0.181	0.209	0.193	0.213	0.242	0.265	0.331	0.220	0.353	0.354	0.210	0.256		0.253
TR	0.121	0.133	0.123	0.141	0.163	0.203	0.277	0.143	0.279	0.288	0.102	0.228	0.190	

Table S5 Bottleneck results for fourteen *Opsariichthys hainanensis* populations based on Wilcoxon's signed-rank test.

		Population													
Model	Wilcoxon test	CH	ND	WQ	LS	LG	LOS	LW	HH	BL	FC	NL	JR	MY	TR
IAM	p (one-tail for H excess)	**0.001**	**0.017**	**0.017**	**0.007**	**0.039**	0.425	0.064	**0.039**	0.396	0.116	0.211	0.604	0.454	**0.046**
TPM	p (one-tail for H excess)	0.133	0.633	0.212	0.810	0.575	0.765	0.367	0.689	0.849	0.259	0.765	0.866	0.740	0.395
SMM	p (one-tail for H excess)	0.968	1.000	0.998	0.998	0.883	0.998	0.633	0.999	0.982	0.311	0.993	0.994	0.945	0.740
Allele frequency distribution		L-shaped													

Values in boldface type are significant at $p<0.05$.

IAM: infinite allele model; TPM: two-phase mutation model; SMM: stepwise mutation model.

Table S6 The distribution information of the haplotypes in *Opsariichthys hainanensis*

Population (Abbreviation)	Mitochondrial DNA (cyt *b* gene and d-loop region)	Nuclear DNA (RAG1)
Changhua River (CH)	H15 - H33	**HN05**, HN11, **HN12**, HN13 - HN15
Nandu River (ND)	**H06** - **H08**, **H10**, **H38**, **H64**, H80 - H85	**HN01**, **HN02**, **HN05**, **HN06**, **HN09**, **HN16**, HN41 - HN47
Wanqan River (WQ)	**H64**, H99 - H111	**HN01**, **HN02**, **HN05**, **HN06**, **HN09**, **HN16**, **HN25**, **HN27**, HN56 - HN58
Lingshui River (LS)	H70, **H71** - H74	**HN01**, **HN02**, **HN05**, **HN06**, **HN09**, HN32 - **HN34**, HN35, HN36
Longgun River (LG)	H63, **H64**, H65	**HN01**, **HN05**, **HN09**, HN23 - **HN25**, HN26, **HN27**, HN28
Longshou River (LOS)	**H66**, H67, **H68**, H69	**HN02**, **HN05**, HN29 - HN31
Longwei River (LW)	**H66**, **H68**, H75	**HN05**, HN37
Tengqiao River (TQ)	**H71**, H92, H93	**HN02**, **HN05**, **HN34**, HN51
Wanglou River (WL)	H97, H98	**HN01**, **HN05**, **HN12**, HN54, HN55
Baisha River (BS)	H11 - H14	**HN05**, **HN09**, HN10
Zhubi River (ZB)	H112 - H117	**HN06**, **HN21**, HN59
Chunjiang River (CJ)	**H07**, H34 - H37, **H38,** H39 - H41	**HN01**, **HN05**, **HN07**, **HN16**, HN17
Beimen River (BM)	**H06** - **H08**, H09, **H10**	**HN01**, **HN02**, **HN05** - **HN07**, HN08
Wenchang River (WC)	H95, H96	**HN01**, **HN06**
Red River (HH)	H45 - H57	**HN01**, HN20
Beilun River (BL)	H01 - H05	**HN01**, **HN02**, HN03 - **HN05**
Fangheng River (FC)	H42 - H44	**HN05**, HN18, HN19
Nanliu River (NL)	H86 - H91	**HN01**, **HN02**, **HN05**, **HN06**, HN48 - HN50
Jian River (JR)	H58 - H62	**HN06**, **HN21**, HN22
Moyang River (MY)	H76 - H79	**HN01**, HN38 - HN40
Tan River (TR)	H94	**HN01**, **HN05**, HN52, HN53

Bold text means shared haplotype.
